# Promotion of colorectal cancer by transcription factor BHLHE40 involves upregulation of *ADAM19* and *KLF7*

**DOI:** 10.3389/fonc.2023.1122238

**Published:** 2023-02-20

**Authors:** Yuan Sui, Hanlin Jiang, Collyn M. Kellogg, Sangphil Oh, Ralf Janknecht

**Affiliations:** ^1^ Department of Pathology, University of Oklahoma Health Sciences Center, Oklahoma City, OK, United States; ^2^ Department of Cell Biology, University of Oklahoma Health Sciences Center, Oklahoma City, OK, United States; ^3^ Stephenson Cancer Center, Oklahoma City, OK, United States

**Keywords:** colorectal cancer, gene transcription, ADAM19, KLF7, BHLHE40, RNA sequencing, histone demethylase

## Abstract

BHLHE40 is a transcription factor, whose role in colorectal cancer has remained elusive. We demonstrate that the *BHLHE40* gene is upregulated in colorectal tumors. Transcription of *BHLHE40* was jointly stimulated by the DNA-binding ETV1 protein and two associated histone demethylases, JMJD1A/KDM3A and JMJD2A/KDM4A, which were shown to also form complexes on their own and whose enzymatic activity was required for *BHLHE40* upregulation. Chromatin immunoprecipitation assays revealed that ETV1, JMJD1A and JMJD2A interacted with several regions within the *BHLHE40* gene promoter, suggesting that these three factors directly control *BHLHE40* transcription. BHLHE40 downregulation suppressed both growth and clonogenic activity of human HCT116 colorectal cancer cells, strongly hinting at a pro-tumorigenic role of BHLHE40. Through RNA sequencing, the transcription factor KLF7 and the metalloproteinase ADAM19 were identified as putative BHLHE40 downstream effectors. Bioinformatic analyses showed that both *KLF7* and *ADAM1*9 are upregulated in colorectal tumors as well as associated with worse survival and their downregulation impaired HCT116 clonogenic activity. In addition, ADAM19, but not KLF7, downregulation reduced HCT116 cell growth. Overall, these data have revealed a ETV1/JMJD1A/JMJD2A→BHLHE40 axis that may stimulate colorectal tumorigenesis through upregulation of genes such as *KLF7* and *ADAM19*, suggesting that targeting this axis represents a potential novel therapeutic avenue.

## Introduction

The DNA-binding protein ETS variant 1 (ETV1; previously also known as ER81) is a downstream effector of signaling proteins such as RAS and HER2/EGFR2 that induce posttranslational modifications on ETV1 and thereby control its transcriptional activity (1–7). This seems to be important not only for normal development and homeostasis, but also during tumorigenesis (8, 9). The most studied association between ETV1 and malignancy pertains to prostate tumors, where ETV1 can be overexpressed due to chromosomal translocations (10). Mimicking this overexpression in mouse prostates led to neoplastic transformation, proving that ETV1 overexpression can indeed be a driver of tumorigenesis (11–14). Likewise, ETV1 has recently been implicated in colorectal cancer development (15, 16), yet the underlying mechanisms have remained unclear.

Transcriptomic studies on human HCT116 colorectal cancer cells pointed at potential downstream effectors of ETV1, including the basic helix-loop-helix family member E40 (BHLHE40; also known as BHLHB2, DEC1, SHARP2 or STRA13) (16). This DNA-binding transcription factor has pleiotropic functions. For instance, BHLHE40 modulates the circadian rhythm that likely involves its interaction with the seminal clock protein BMAL1 (17–19), affects immune responses and establishment of autoimmune disorders (20), enhances hippocampal synaptic plasticity (21), or attenuates lipid consumption and thereby facilitates high-fat diet-induced obesity (22). However, BHLHE40 is not essential, as respective homozygous knockout mice were viable and fertile and only displayed lymphoproliferative defects at later age with variable penetrance depending on the genetic background (23, 24).

The role of BHLHE40 in cancer is complex. Depending on the tissue and the stage of the disease, BHLHE40 has been implicated to promote or suppress tumorigenesis (25). An earlier study suggested that BHLHE40 is upregulated at both the mRNA and protein level in colon tumors, but this was based on a very small sample number of four (26). A more recent report analyzed BHLHE40 protein levels in ten colorectal tumors by immunohistochemistry and claimed overexpression, but no quantification or statistical analysis was provided (27). Moreover, these authors employed whole-body *Bhlhe40* knockout mice and demonstrated that colon carcinogenesis induced by azoxymethane/dextran sulfate was compromised upon BHLHE40 ablation (27). However, chemically induced colon carcinogenesis is highly dependent on inflammation and the immune system. Given that BHLHE40 expression affects the T cell repertoire and thus likely modulates inflammatory responses in colorectal cancer (28), it is possible that BHLHE40 ablation in the immune cells rather than the colon epithelium antagonized azoxymethane/dextran sulfate-induced cell transformation and thereby colon cancer development.

Here, we provide evidence that BHLHE40 is needed for efficient growth and clonogenic activity of colorectal cancer cells. In addition, we demonstrate that *BHLHE40* upregulation can be brought about by ETV1 together with two interacting epigenetic regulators, JMJD1A (also known as lysine demethylase (KDM) 3A) and JMJD2A (also known as KDM4A), which are histone demethylases (29–32). Finally, we uncovered how BHLHE40 may promote colon tumorigenesis by regulating the expression of various genes, including *KLF7* and *ADAM19*.

## Materials and methods

### Virus production and infection

Retrovirus was produced according to standard procedures (33). Viral supernatant was then used to infect 2-3 times HCT116 colorectal cancer cells (CCL-247; American Type Culture Collection), followed by selection with 1-2 µg/ml puromycin (34). To validate effectiveness of viral infection, protein extracts were prepared from stably transduced cells and assessed by Western blotting (35). Rabbit polyclonal antibodies against BHLHE40 [Novus, NB100-1800), ETV1 (#959 generated by our laboratory (36)], JMJD1A (Novus, NB100-77282), JMJD2A (Bethyl, A300-861A) and actin (Sigma, A2066) were used as primary antibodies. The following shRNAs were utilized: shBHLHE40#1 (GGAGACCTACAAATTGCCG), shBHLHE40#2 (GAACCGGATGCAGCTTTCG), shBHLHE40#4 (GAGCTCAGACTCAGAGGAA), shADAM19#2 (GTTGTACTGTGAACTGGAA), shADAM19#3 (GAGTGTGACTGTGGAGAAG), shKLF7#2 (GGAGTCTACAGTCCTTACT) and shKLF7#6 (GCCTTATAAGTGCTCATGG). The shRNA sequences for ETV1, JMJD1A and JMJD2A have been published before (16, 36, 37).

### RT-PCR and RNA sequencing

RNA was isolated with Trizol (38) and further purified with the RNeasy Mini kit (Qiagen, #74104). Utilizing random p(dN)_6_ primers, RNA was reverse transcribed (GoScript Reverse Transcription; Promega, #A5000) and then examined in real-time with the AccuPower 2xGreenstar qPCR kit (Bioneer, #K-6251) in a BioRad light cycler according to the manufacturer’s recommendations. Sequences of RT-PCR primers are provided in **Supplementary Table 1**. RNA sequencing was performed by Novogene and analysis of data done as described (39).

### Chromatin immunoprecipitation assays

HCT116 cells were treated with formaldehyde and chromatin immunoprecipitations then performed as described (40). Antibodies used were control rabbit IgG (Santa Cruz, sc-2027), rabbit polyclonal anti-JMJD1A antibodies (Novus Biologicals, NB100-77282), anti-JMJD2A antibodies (Bethyl, A300-861A) or previously reported (36) anti-ETV1 antibodies. *BHLHE40* promoter fragments were amplified with nested PCR (16) and then visualized through ethidium bromide staining (41) after agarose gel electrophoresis (42). Primers used for amplification are listed in **Supplementary Table 2**.

### Luciferase assays

HCT116 cells were grown in 12-wells and transiently transfected with the help of 2 µg polyethylenimine (43). A total of 0.25 µg luciferase reporter plasmid, 0.9 µg pBluescript KS^+^, and 0.1 µg of empty vector pEV3S or ETV1 expression vector were used for each transfection when comparing pBL2-Basic to BHLHE40-luc, while 0.1 µg BHLHE40 luciferase reporter plasmid, 0.9 µg pBluescript KS^+^, 80 ng empty vector pEV3S or ETV1 expression vector, and 60 ng of empty vector pEV3S or indicated JMJD expression vector were employed otherwise. Eight hours afterwards, cells were washed with phosphate-buffered saline, and cell extracts were prepared 1.5 days later (44). Luciferase activity was then determined in a luminometer as described (45).

### Coimmunoprecipitations

Human embryonic kidney 293T cells (CRL-3216; American Type Culture Collection) were cultured in poly-*L*-lysine coated 6-cm plates (46). Upon reaching 25% confluency, cells were transiently transfected by the calcium phosphate coprecipitation method (47). Two days after transfection, cell extracts were prepared (48) and immunoprecipitations performed as previously described (49). Precipitated proteins were run on SDS polyacrylamide gels (50), transferred to polyvinylidene fluoride membrane (51), and then challenged with indicated antibodies (52). After incubation with corresponding secondary antibodies coupled to horseradish peroxidase (53), signals were revealed by chemiluminescence (54) and exposure to film (55). Likewise, endogenous proteins were coimmunoprecipitated from human HCT116 colorectal cancer cells, utilizing control rabbit IgG (Santa Cruz, sc-2027) or rabbit polyclonal anti-JMJD1A antibodies (Bethyl, A301-538A) for immunoprecipitation followed by Western blotting with anti-JMJD2A antibodies (Bethyl, A300-861A).

### Growth assays

Transduced HCT116 cells were seeded into 96-well plates at a density of 2800 cells/well and their growth measured as described (56). For clonogenic assays, 2000 cells were seeded into 6-well plates, grown for approximately 10 days, and then stained with crystal violet (57).

## Results

### Regulation of *BHLHE40* transcription by ETV1, JMJD1A and JMJD2A

**Figure 1:**
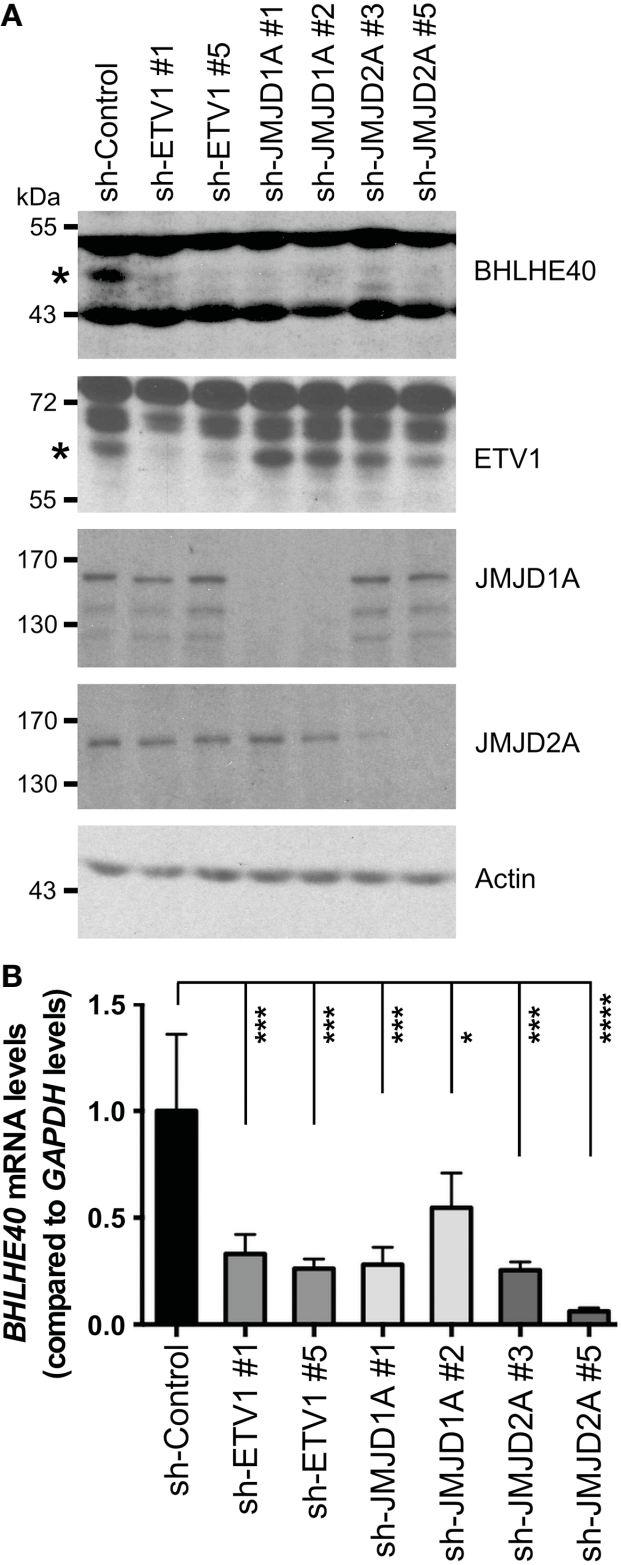
Regulation of BHLHE40 expression by ETV1 and associated JMJD cofactors. **(A)** Downregulation of ETV1, JMJD1A or JMJD2A with respective shRNAs in HCT116 cells reduced BHLHE40 protein levels. Asterisks mark BHLHE40 or ETV1, while other bands are unspecific in the respective blots. **(B)** Corresponding quantitative RT-PCR examining *BHLHE40* mRNA levels. Shown are means with standard deviations (n=3). One-way ANOVA with *post hoc* Dunnett’s multiple comparisons test. *P < 0.05; ***P < 0.001; ****P < 0.0001.

Previously, we performed RNA sequencing on ETV1-depleted human HCT116 colorectal cancer cells and found downregulation of *BHLHE40* mRNA (16). To corroborate this finding, we performed Western blotting with HCT116 cells in which ETV1 was downregulated with two different shRNAs. And we expectedly observed that BHLHE40 protein levels became reduced upon ETV1 ablation (**Figure 1A**). Likewise, we downregulated the histone demethylase JMJD1A, which is capable of binding to ETV1 and also reportedly affects *BHLHE40* transcription (16), and observed concurrent decreases of JMJD1A and BHLHE40 protein levels (**Figure 1A**). Like JMJD1A, the histone demethylase JMJD2A also directly binds to ETV1 (37), prompting us to assess if *BHLHE40* would similarly be regulated by JMJD2A. And indeed, BHLHE40 was downregulated at both the protein and mRNA level upon expression of two different JMJD2A shRNAs, and the degree of this downregulation was similar to that observed with ETV1 or JMJD1A shRNAs (**Figures 1A, B**). These data imply that *BHLHE40* gene transcription can be jointly regulated by ETV1, JMJD1A and JMJD2A.

**Figure 2:**
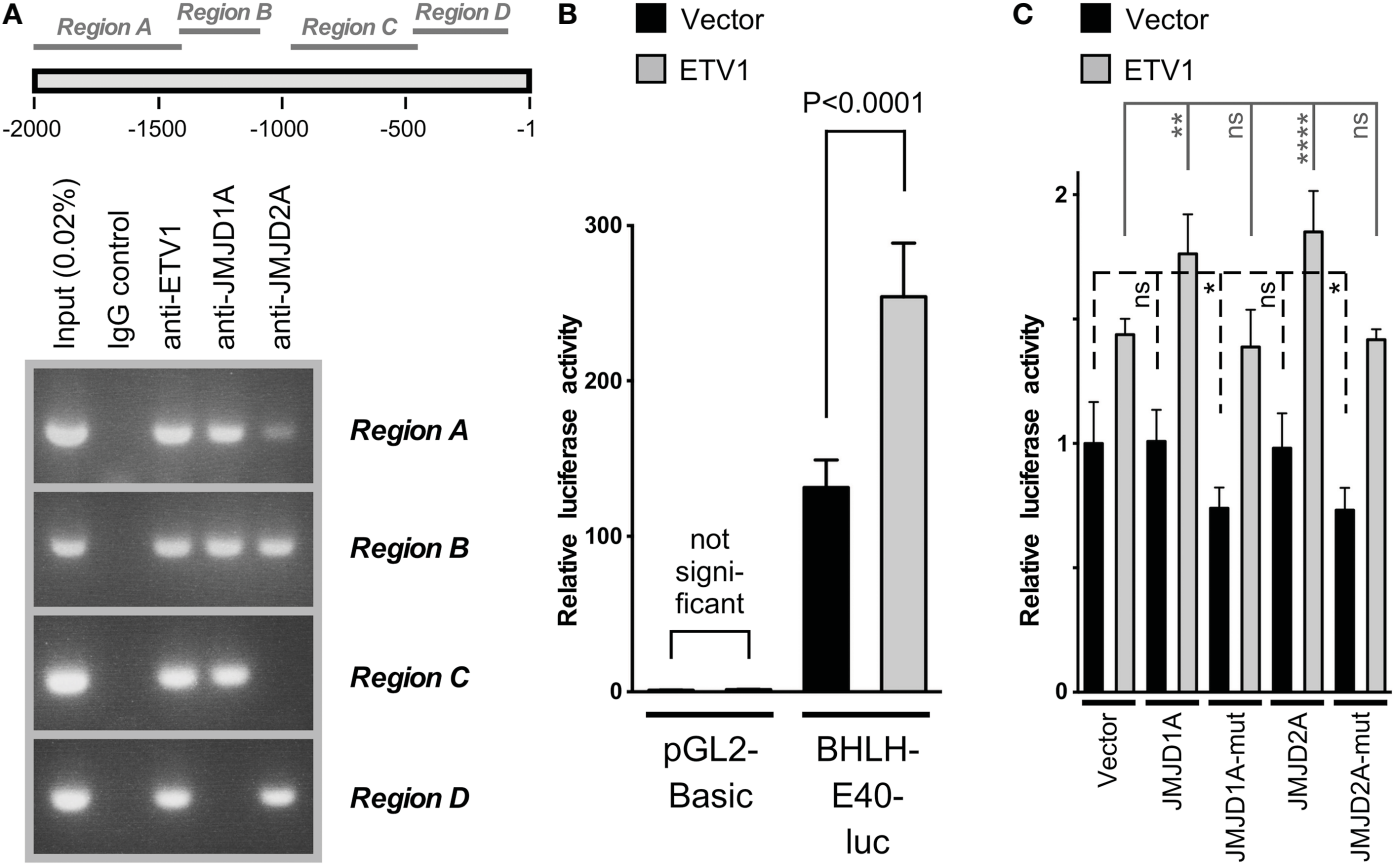
Analysis of the *BHLHE40* gene promoter in HCT116 cells. **(A)** Chromatin immunoprecipitation assay. Four different regions (see scheme on top) of the *BHLHE40* promoter were amplified after immunoprecipitation with indicated antibodies. **(B)** Activation of the (-2000/+50) *BHLHE40* promoter luciferase reporter construct or the parental vector pGL2-Basic upon ETV1 overexpression. Shown are means with standard deviations (n=4). One-way ANOVA with *post hoc* Tukey’s multiple comparisons test. **(C)** Similar, joint stimulation of the (-2000/+50) *BHLHE40* promoter luciferase activity by ETV1 and either JMJD1A or JMJD2A; “mut” indicates catalytically inactive mutants of JMJD1A (H1120A/D1122G) or JMJD2A (H188A). Shown are means with standard deviations (n=6). Statistical significance was determined by one-way ANOVA with *post hoc* Tukey’s multiple comparisons test. *P < 0.05; **P < 0.01; ****P < 0.0001; ns, not significant.

Consistently, chromatin immunoprecipitation assays revealed that all three of these proteins bound to several regions within the human *BHLHE40* gene promoter (**Figure 2A**). Notably, the *BHLHE40* promoter contains numerous potential ETV1 binding sites throughout its length (**Supplementary Figure 1**), providing a possible explanation why ETV1 bound to all four tested regions of the *BHLHE40* promoter. Further, we cloned the *BHLHE40* promoter in front of a luciferase gene and then determined that ETV1 was capable of activating this reporter construct (**Figure 2B**). In contrast, neither ectopic JMJD1A nor ectopic JMJD2A alone activated the *BHLHE40* luciferase reporter gene, but either one of these histone demethylases cooperated with ETV1 in doing so (**Figure 2C**). In contrast, catalytically inactive mutants of JMJD1A and JMJD2A were unable to cooperate with ETV1, and their expression alone even suppressed the *BHLHE40* luciferase reporter gene (**Figure 2C**), possibly by competing with and thus precluding endogenous JMJD1A and JMJD2A to cooperate with endogenous ETV1. Overall, these data suggest that ETV1, JMJD1A and JMJD2A are jointly involved in the upregulation of the *BHLHE40* gene promoter. In further support of this notion, *BHLHE40* mRNA levels are positively correlated with *ETV1*, *JMJD1A* and *JMJD2A* in the 592 colorectal adenocarcinomas represented in The Cancer Genome Atlas (TCGA; **Supplementary Figure 2**).

**Figure 3:**
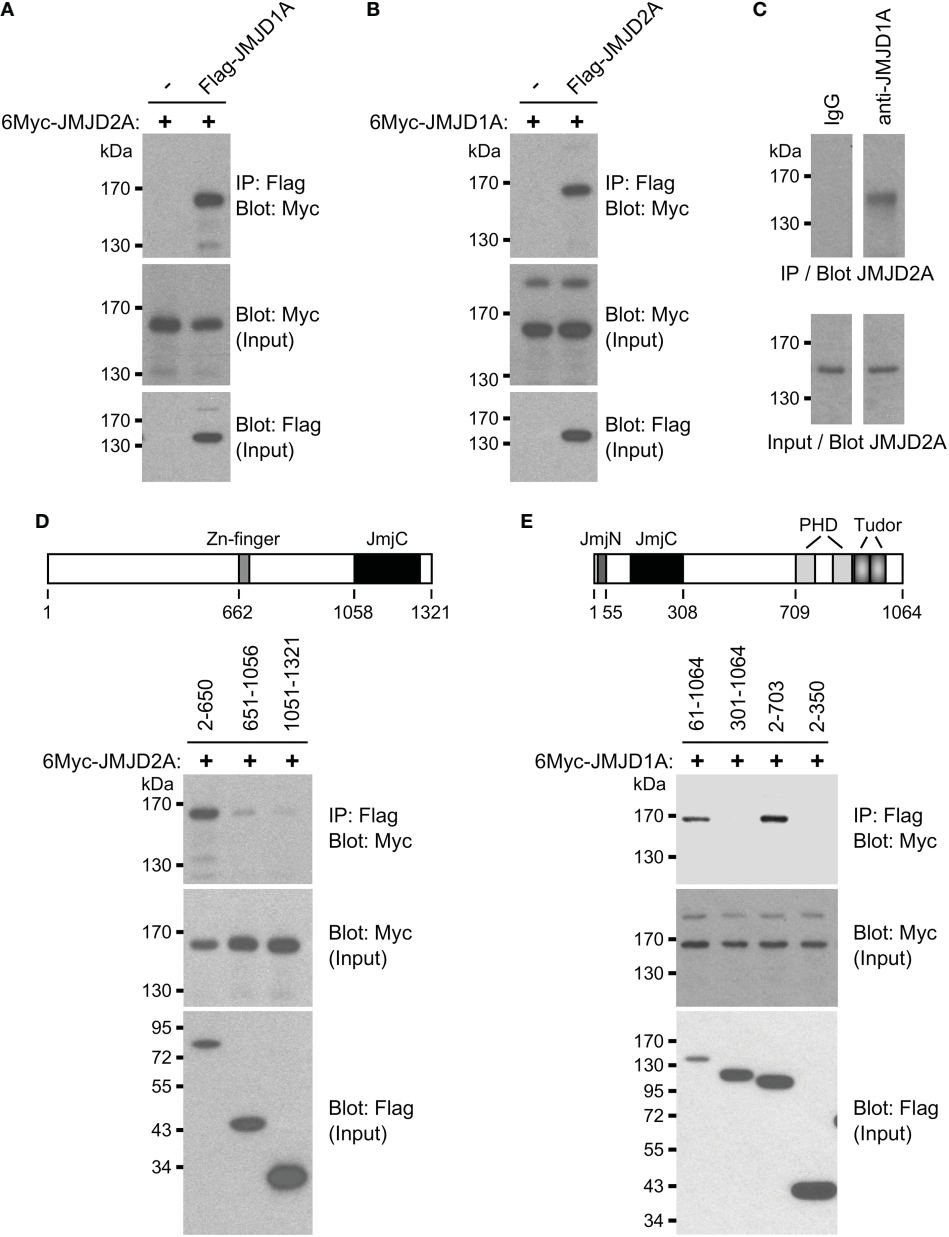
Complex formation between JMJD1A and JMJD2A. **(A)** Coimmunoprecipitation experiment with 6Myc-JMJD2A and Flag-JMJD1A in 293T cells. The top panel shows immunoprecipitation (IP) with anti-Flag antibodies followed by anti-Myc Western blotting, while the bottom two panels show input levels for 6Myc-JMJD2A and Flag-JMJD1A. **(B)** Likewise, interaction between 6Myc-JMJD1A and Flag-JMJD2A. **(C)** Endogenous coimmunoprecipitation of JMJD2A with JMJD1A in HCT116 cells. Either control IgG or anti-JMJD1A antibody was used for immunoprecipitation. **(D)** Coimmunoprecipitations of indicated Flag-tagged JMJD1A amino acids with 6Myc-JMJD2A. Top shows a sketch of JMJD1A. **(E)** Coimmunoprecipitation of 6Myc-JMJD1A with indicated Flag-tagged JMJD2A amino acids. Top shows a sketch of JMJD2A.

Because ETV1 can bind to both JMJD1A and JMJD2A (16, 37), their joint regulation of *BHLHE40* transcription may involve the formation of a ternary complex of all these three factors. This would be consistent with the fact that all of these three transcription factors interacted with the same regions A and B of the *BHLHE40* gene promoter in our chromatin immunoprecipitation experiments (**Figure 2A**). This additionally prompted us to examine if JMJD1A and JMJD2A would also form complexes with each other and not only with ETV1. To this end, we coexpressed Flag-tagged JMJD1A and Myc-tagged JMJD2A, performed immunoprecipitations with anti-Flag antibodies and probed with anti-Myc antibodies if JMJD2A would coimmunoprecipitate. And this was the case (**Figure 3A**), and this interaction between JMJD1A and JMJD2A could be confirmed by switching the tags in another coimmunoprecipitation experiment (**Figure 3B**) as well as upon assessing the complex formation between endogenous JMJD1A and endogenous JMJD2A in HCT116 cells (**Figure 3C**). We then determined that the N-terminal half of JMJD1A (amino acids 2-650), which does not contain the catalytic JmjC domain nor a Zn-finger also required for catalytic activity, primarily mediated the interaction with JMJD2A (**Figure 3D**). Deletion of the N-terminal JmjN domain that is required for the catalytic activity of JMJD2A did not abolish binding to JMJD1A (see 61-1064 in **Figure 3E**), but further deletion of the catalytic core JmjC domain did (see 301-1064). While this shows that the JmjC domain of JMJD2A is required for binding to JMJD1A, it is not sufficient since JMJD2A amino acids 2-350 did not bind to JMJD1A (**Figure 3E**). But JMJD2A amino acids 2-703 interacted with JMJD1A, indicating that the JMJD2A JmjC domain together with about 400 amino acids C-terminal of it jointly mediate the interaction with JMJD1A.

### Stimulation of HCT116 cells by BHLHE40

**Figure 4:**
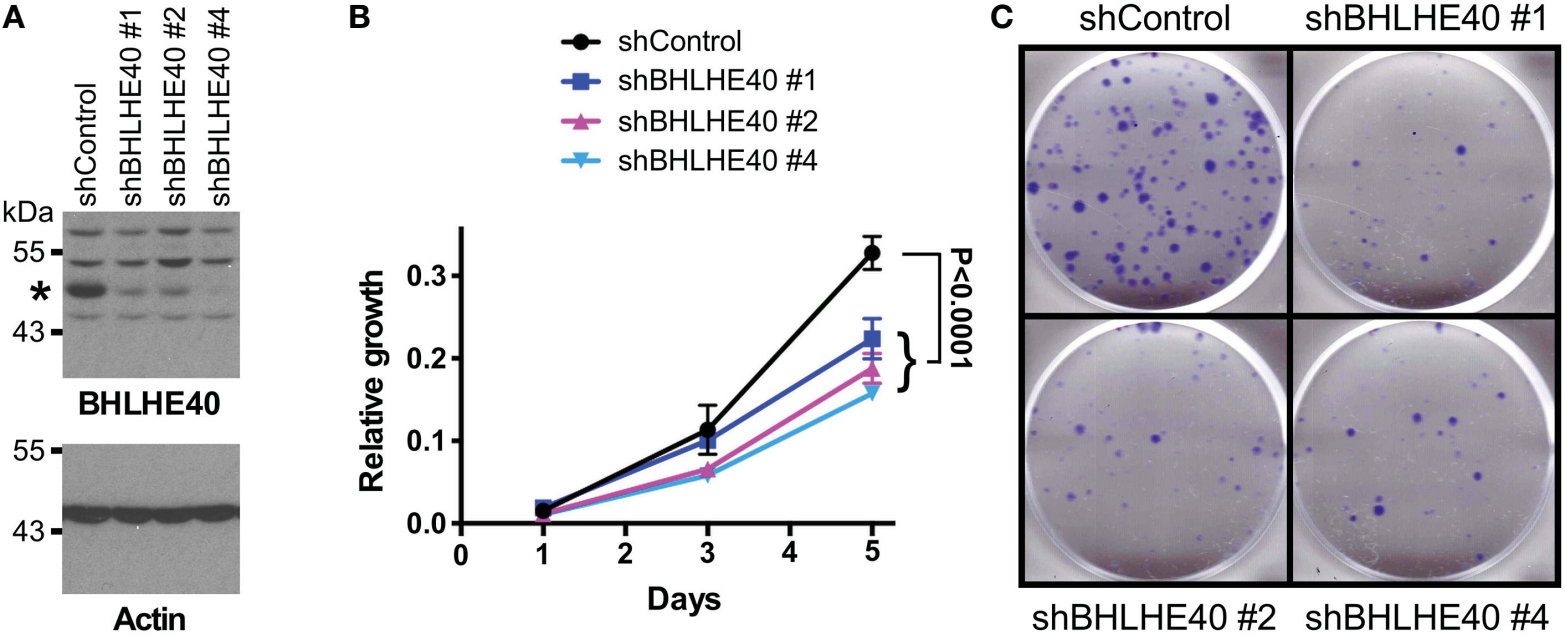
BHLHE40 promotes HCT116 growth. **(A)** Downregulation of BHLHE40 with three different shRNAs. Shown are BHLHE40 and actin Western blots. Asterisk marks BHLHE40. **(B)** Cell growth assay. Shown are means with standard deviations (n=3). Two-way ANOVA with *post hoc* Tukey’s multiple comparisons test. **(C)** Corresponding clonogenic assays with representative images.

**Figure 5:**
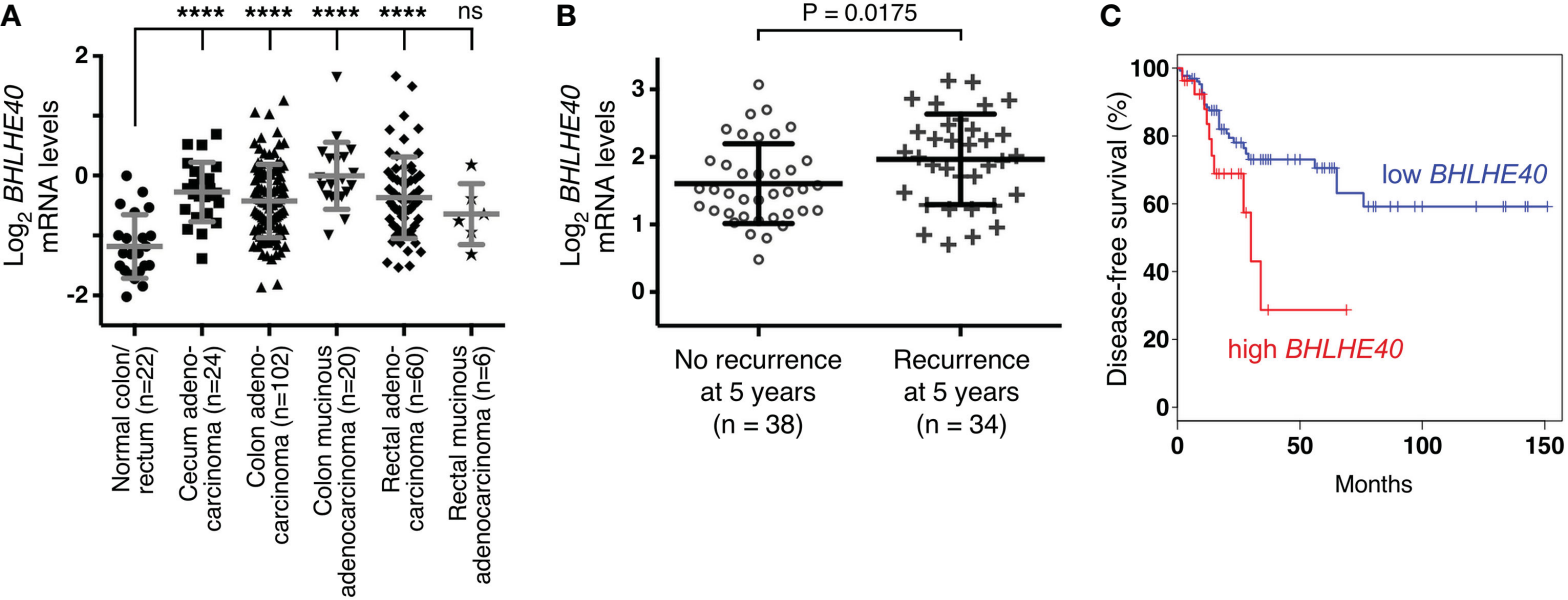
Expression of *BHLHE40* in colorectal tumors. **(A)** *BHLHE40* mRNA levels (reporter A_23_P57838; median-centered ratio) in TCGA microarray experiments (63). One-way ANOVA (Dunnett’s multiple comparisons test); ****P < 0.0001; ns, not significant. **(B)** Association of *BHLHE40* mRNA levels with recurrence of colorectal carcinomas 5 years after diagnosis (data from reference (64); reporter 201169_s_at; median-centered ratio); unpaired, two-tailed t test. **(C)** Kaplan-Meier survival curve for patients with colorectal adenocarcinoma comparing high (90% cutoff, n=27) with low (50% cutoff, n=135) *BHLHE40* mRNA expression. Analysis was done with GEPIA (http://gepia.cancer-pku.cn/index.html). P=0.028 (Logrank test).

Ablation of ETV1 (16), JMJD1A (58–60) or JMJD2A (61) alone reportedly suppressed growth or clonogenic activity of HCT116 cells, indicating that all three factors are required for the full oncogenic potential of these colorectal cancer cells. Hence, we tested whether their joint target gene, *BHLHE40*, would also promote the growth and/or clonogenic activity of HCT116 cells. To this end, we downregulated BHLHE40 with three different shRNAs and observed that this resulted in both reduced cell growth and clonogenic activity (**Figure 4**). While this manuscript was under preparation, another study similarly showed that a BHLHE40 siRNA was capable of reducing HCT116 cell growth (62). These data implicate that BHLHE40 in its own right is a tumor promoter in colorectal cancer. In agreement with this, analysis of published microarray data (63, 64) revealed that *BHLHE40* mRNA levels are upregulated in colorectal tumors (**Figure 5A** and **Supplementary Figures 3A-D**) and may be a predictor of disease recurrence (**Figure 5B** and **Supplementary Figures 3E, F**) and disease-free survival (**Figure 5C**). Altogether, these data indicate that upregulation of *BHLHE40* in human colorectal tumors may promote their growth and aggressiveness.

### Influence of BHLHE40 on the transcriptome

**Figure 6:**
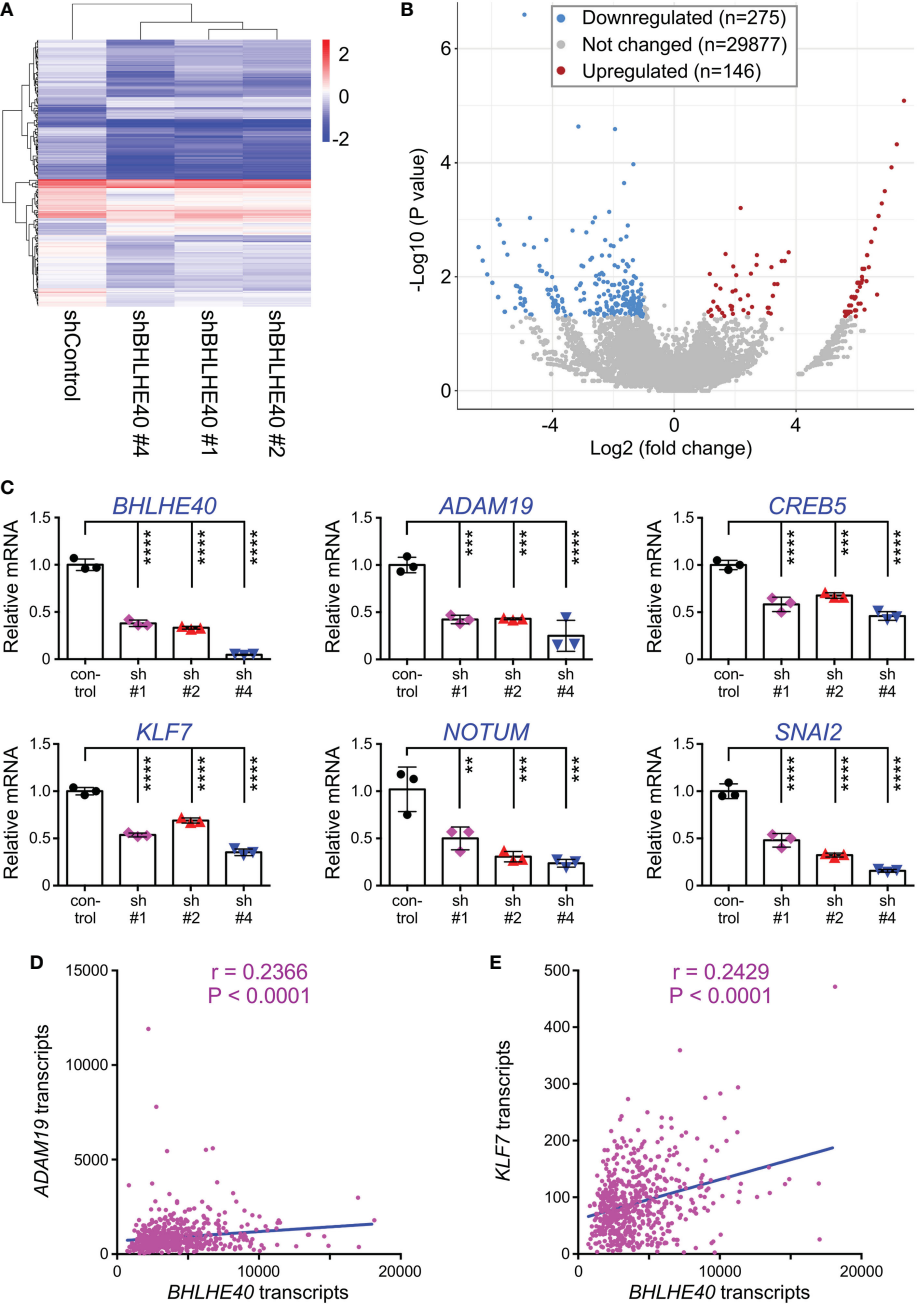
Transcriptome changes upon BHLHE40 downregulation. **(A)** Heat map of transcript changes between control HCT116 cells and those with downregulation of BHLHE40. **(B)** Volcano plot showing average P values versus average fold changes. Only genes with absolute transcript level changes of greater than 2 and P<0.05 were considered up- or downregulated. **(C)** Validation by quantitative RT-PCR for indicated transcripts that were found downregulated upon expression of BHLHE40 shRNAs. All mRNA levels were normalized to *GAPDH*. One-way ANOVA (Dunnett’s multiple comparisons test; n=3); **P < 0.01; ***P < 0.001; ****P < 0.0001. **(D)** Correlation of *BHLHE40* mRNA levels with those of *ADAM19* in 592 colorectal adenocarcinomas. Data were derived from the TCGA PanCancer Atlas (RSEM values; batch normalized from Illumina HiSeq_RNASeqV2); r=Spearman correlation coefficient. **(E)** Analogous for *KLF7*.

To gain mechanistic insights how BHLHE40 could affect colon cancer cells, we performed RNA sequencing on HCT116 cells that expressed three different BHLHE40 shRNAs. We found that this caused the down-/upregulation of 275/146 genes (**Figures 6A, B**). The fact that only 1.4% of all transcripts were significantly altered implies that BHLHE40 has a strong selectivity in its function as a transcriptional regulator. We then validated the RNA sequencing data by quantitative RT-PCR with a small selection of the differentially expressed genes (all downregulated in RNA sequencing data; **Figure 6C**). Amongst those, we selected *ADAM19*, which encodes for a metalloproteinase, and *KLF7*, which encodes for a DNA-binding transcription factor, for further analysis, because their expression was positively correlated with *BHLHE40* in 592 colon adenocarcinomas (**Figures 6D, E**) and little was known about their role in colon cancer.

### Role of *KLF7* and *ADAM19* in colorectal cancer cells

**Figure 7:**
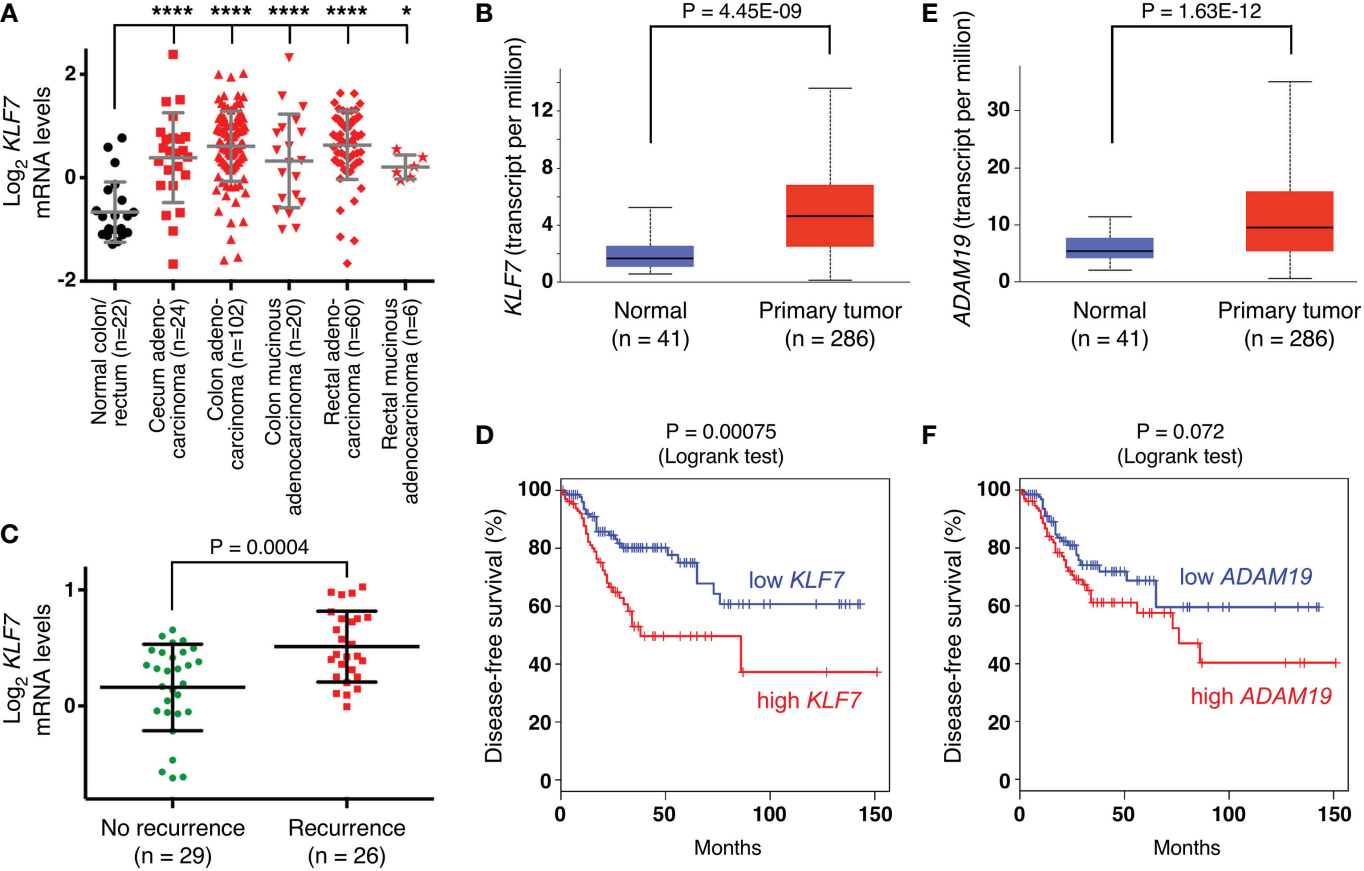
Expression of *KLF7* and *ADAM19* in colorectal tumors. **(A)** *KLF7* mRNA levels (reporter A_23_P67978; median-centered ratio) in TCGA microarray experiments (63). One-way ANOVA (Dunnett’s multiple comparisons test); *P < 0.05; ****P < 0.0001. **(B)** *KLF7* transcripts determined by RNA sequencing in TCGA colorectal adenocarcinomas (box plot; unpaired, two-tailed t test). Analysis was done with UALCAN (http://ualcan.path.uab.edu). **(C)** Association of *KLF7* mRNA levels with recurrence of colorectal carcinomas 5 years after diagnosis (data from reference (65); reporter 204334_at; median-centered ratio); unpaired, two-tailed t test. **(D)** Disease-free survival for patients with colorectal adenocarcinoma comparing high (median cutoff, n=135) with low (median cutoff, n=135) *KLF7* mRNA expression. Analysis was done with GEPIA (http://gepia.cancer-pku.cn/index.html). **(E)** As in panel **(B)** for *ADAM19*. **(F)** As in panel D for *ADAM19*.

First, we determined in published (63) microarray data that *KLF7* mRNA is significantly upregulated in various subtypes of colorectal cancer (**Figure 7A**). Likewise, analysis of public RNA sequencing data revealed upregulation of *KLF7* mRNA in colorectal adenocarcinomas at the primary tumor site (**Figure 7B**). Moreover, we found a significant association of *KLF7* mRNA with disease recurrence in another published (65) microarray data set (**Figure 7C**). Further, high *KLF7* levels are associated with reduced disease-free survival (**Figure 7D**). Altogether, these data suggest that KLF7 may promote colon tumorigenesis and may be a predictor of worse outcome. Similarly, we found that *ADAM19* is significantly overexpressed in colorectal adenocarcinomas (**Figure 7E**), corroborating a previous study comparing a small number (n=14) of colorectal tumors to normal tissue (66). And high *ADAM19* expression appears to also be predictive of worse disease-free survival, although this does not reach statistical significance (**Figure 7F**), suggesting that ADAM19 exerts pro-tumorigenic functions in colon cancer.

**Figure 8:**
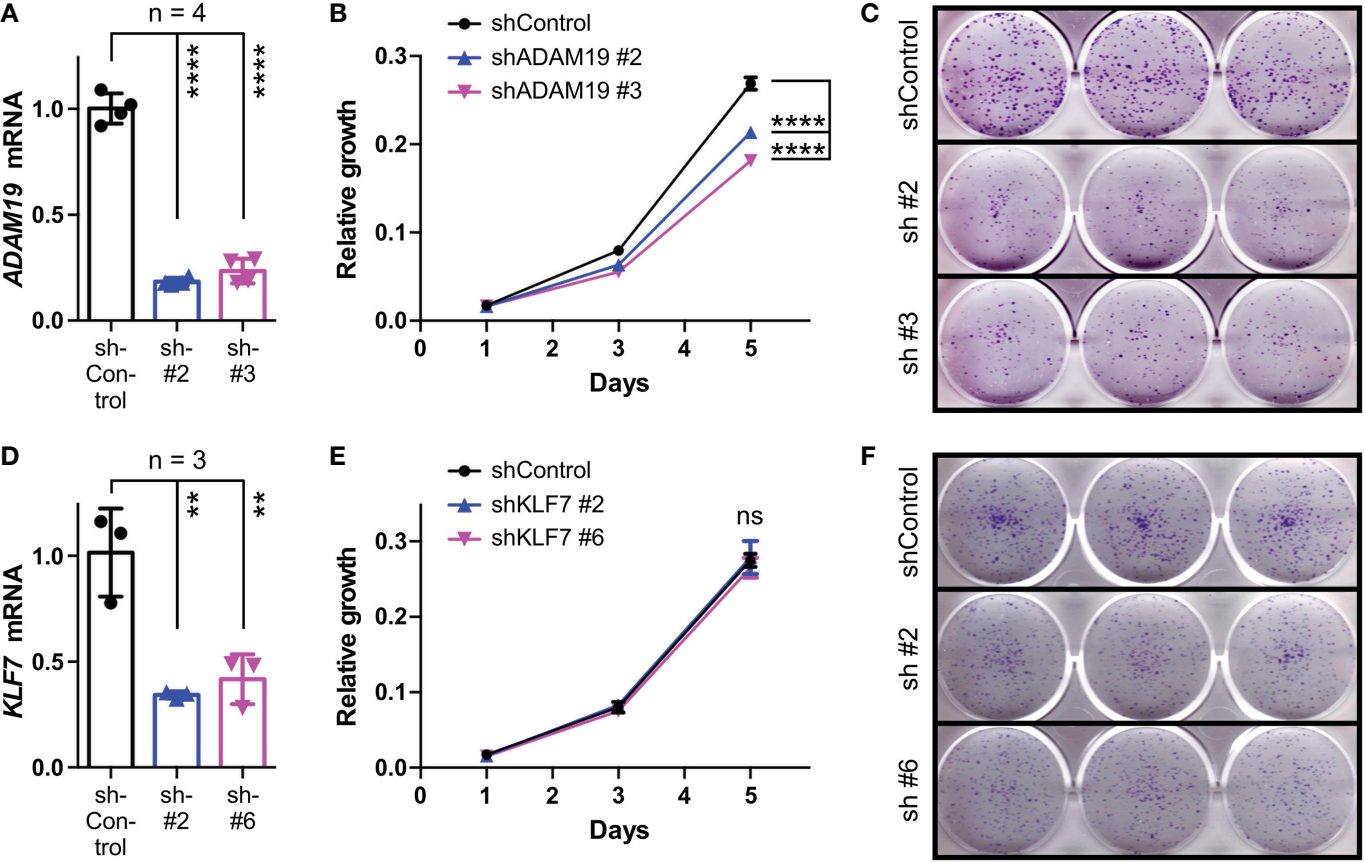
Role of KLF7 and ADAM19 for HCT116 cell growth and clonogenicity. **(A)** Downregulation of ADAM19 with two different shRNAs. Shown are *ADAM19* mRNA levels normalized to *GAPDH* levels (means with standard deviations). One-way ANOVA with *post hoc* Tukey’s multiple comparisons test. **(B)** Impact of ADAM19 downregulation on cell growth. Shown are means with standard deviations (n=3). Two-way ANOVA with *post hoc* Tukey’s multiple comparisons test. **(C)** Clonogenic assays upon ADAM19 downregulation; shown are representative images. **(D-F)** Analogous to panels A-C for KLF7. **P < 0.01; ****P < 0.0001; ns, not significant.

We then tested how downregulation of *KLF7* or *ADAM19* would affect HCT116 cells. Using two independent shRNAs, we achieved efficient *ADAM19* downregulation (**Figure 8A**). Both of these ADAM19 shRNAs caused a significant decrease in HCT116 cell growth and clonogenic activity (**Figures 8B, C**), which is similar to what we observed upon BHLHE40 downregulation (see **Figure 4**). In contrast, downregulation of KLF7 did not affect HCT116 cell growth, but still led to a reduction in clonogenic activity (**Figures 8D-F**), albeit less so than compared to ADAM19 downregulation. These data suggest that KLF7 and especially ADAM19 are downstream effectors of BHLHE40.

## Discussion

In the present study, we have discovered that *BHLHE40* transcription is jointly stimulated by ETV1, JMJD1A and JMJD2A, that BHLHE40 can stimulate growth and clonogenic activity of HCT116 colorectal cancer cells, that *KLF7* and *ADAM19* are regulated by BHLHE40, and that KLF7 and ADAM19 are in their own right potential promoters of colorectal tumors.

Corroborating previous studies (26, 27), we found that *BHLHE40* is upregulated in colorectal tumors. Importantly, our results demonstrated that high *BHLHE40* levels are correlated with more disease recurrence and reduced survival, implying that BHLHE40 levels can predict disease aggressiveness. Why BHLHE40 is upregulated in colorectal cancer has remained unknown, but our study strongly indicates that this could be due to enhanced activity of ETV1, JMJD1A and/or JMJD2A. Both ETV1 and JMJD1A are reportedly overexpressed in colorectal tumors (15, 16, 58–60), providing one plausible explanation for *BHLHE40* upregulation in this cancer. Since ETV1, JMJD1A and JMJD2A are implicated in many different types of malignancies (8, 29–32), *BHLHE40* upregulation by these three proteins is likely not limited to colorectal tumors. Notably, results from our chromatin immunoprecipitation experiments support the notion that a ternary complex of ETV1, JMJD1A and JMJD2A may bind to the *BHLHE40* promoter in regions A and B (see **Figure 2A**); however, it appears that binary complexes of ETV1/JMJD1A and ETV1/JMJD2A are also involved in *BHLHE40* upregulation, as exclusive binding of respective protein pairs was found in regions C and D, respectively, of the *BHLHE40* gene promoter (see **Figure 2A**). Further, since we demonstrated that JMJD1A and JMJD2A formed complexes by themselves, these two proteins could be recruited together by a DNA-binding transcription factor(s) other than ETV1 to various gene promoters. The joint recruitment of JMJD1A and JMJD2A may be very effective to remove the repressive H3K9me_3/2_ epigenetic promoter marks, because JMJD2A preferentially demethylates H3K9me_3_ to H3K9me_2_, which can then be converted to H3K9me_1_ by JMJD1A (29–32). And the cooperation between JMJD1A and JMJD2A in regulating *BHLHE40* transcription may be relevant to diseases other than cancer, including cardiac hypertrophy and fibrosis that are dependent on BHLHE40 (67, 68) and promoted by JMJD1A and JMJD2A overexpression (69, 70).

BHLHE40 is capable of binding to specific DNA sites and this often leads to transcriptional repression, possibly through recruitment of a histone deacetylase (25, 71–73). However, BHLHE40 can also stimulate gene transcription, but this may in part be through recruitment by other DNA-binding proteins such as STAT3 or SP1 (74, 75). Furthermore, BHLHE40 may influence gene transcription by modulating the formation of chromatin loops through binding CTCF (76). Our transcriptomic analyses in HCT116 colorectal cancer cells showed that downregulation of BHLHE40 led more often to a reduction than an increase of gene transcription, suggesting that BHLHE40 is mostly stimulating transcription of genes, including *KLF7* and *ADAM19*. If this is through direct DNA binding at respective gene promoters or other means remains to be studied.

The transcription factor KLF7 plays important roles in neuronal morphogenesis, which is why its knockout in mice led to neonatal death (77, 78). It also plays important roles in tumorigenesis. For instance, KLF7 has been implicated as a tumor promoter in oral squamous carcinoma (79), pancreatic cancer (80), hepatocellular carcinoma (81) or gastric cancer (82), but its role in colorectal cancer has been elusive. Our data indicate that KLF7 may promote the clonogenic activity of colorectal cancer cells and that KLF7 overexpression may even have prognostic value. Interestingly, KLF7 has been shown to modulate stem cells and lineage differentiation (83, 84), but if KLF7 likewise influences cancer stem cells and how this may relate to colon tumorigenesis remains to be studied.

ADAM19 belongs to a family of metalloproteinases that have various functions. Their proteolytic activity can activate growth factors and cytokines, lead to the shedding of ectodomains of membrane-bound receptors, influence integrin signaling and degrade extracellular matrix components. As such, ADAM proteins can have pleiotropic effects, including regulating cell proliferation, migration and invasion in normal and cancer cells, and are therefore potential targets in cancer therapy (85, 86). This pertains also to ADAM19, which has previously been shown to be overexpressed in brain tumors and implicated as a promoter of invasion (87, 88). Likewise, a previous study (66) and our data strongly suggest that ADAM19 is overexpressed in colorectal cancer and may be a predictor of survival. Overexpression of ADAM19 in colorectal cancer may in part be due to the downregulation of microRNAs targeting ADAM19 (89, 90). Interestingly, overexpression of ADAM10, ADAM17 and ADAM19 together resulted in increased migration and invasion of HCT116 cells, but it remained undefined which of the three ADAMs was responsible (66). Our data show that ADAM19 downregulation decreased HCT116 cell growth and clonogenic activity, which hints at a pro-tumorigenic function of ADAM19 in colorectal cancer.

In our RNA sequencing study, we found that many genes besides *KLF7* and *ADAM19* were, directly or indirectly, regulated by BHLHE40 and might therefore also (partially) mediate BHLHE40’s tumorigenic functions. For instance, the *CREB5* transcription factor gene was downregulated in the BHLHE40 shRNA-treated HCT116 cells, suggesting that BHLHE40 stimulates CREB5 expression. And CREB5 is overexpressed in colorectal tumors and capable of enhancing proliferation, invasion and metastasis of colorectal cancer cells (91, 92). Similarly, *SNAI2*, which encodes for a zinc-finger transcription factor regulating epithelial-mesenchymal transition and is overexpressed in colorectal tumors (93), was positively regulated by BHLHE40, and SNAI2 is capable of stimulating colorectal cancer cell invasion and tumor formation (94–96). NOTUM, which encodes for a palmitoleoyl-protein carboxylesterase being overexpressed in colon tumors and supporting tumor initiation and proliferation (97–100), was also found to be downregulated upon BHLHE40 ablation. This suggests that BHLHE40 acts through multiple effectors to facilitate tumorigenesis.

In conclusion, our data have uncovered a ETV1/JMJD1A/JMJD2A→BHLHE40 axis in colon cancer cells. Antagonizing this axis may potentially suppress colorectal tumor formation, and likewise the inhibition of BHLHE40-regulated genes such as *ADAM19* or *KLF7* may have therapeutic value.

## Data availability statement

The datasets presented in this study can be found in online repositories. The names of the repository/repositories and accession number(s) can be found below: https://www.ncbi.nlm.nih.gov/, PRJNA859517.

## Author contributions

YS, HJ, CK and RJ conceived, designed and performed experiments. YS, HJ, CK, SO and RJ analyzed and interpreted data as well as contributed to the writing of this manuscript. RJ supervised this study. All authors contributed to the article and approved the submitted version.
