# Supplementary material for: Promotion of colorectal cancer by transcription factor BHLHE40 involves upregulation of ADAM19 and KLF7
Source: Front Oncol. 2023 Feb 20;13:1122238. doi: 10.3389/fonc.2023.1122238 (PMC9986587; doi:10.3389/fonc.2023.1122238)
Supplement: Supplementary file 1 [file DataSheet_1.pdf]

Supplementary Material

Promotion of colorectal cancer by transcription factor BHLHE40 involves upregulation of *ADAM19* and *KLF7*

*Yuan Sui, Hanlin Jiang, Collyn M. Kellogg, Sangphil Oh, Ralf Janknecht**

*** Correspondence:** ralf-janknecht@ouhsc.edu

SUPPLEMENTARY TABLES

Gene	Forward primer (5' to 3')	Reverse primer (5' to 3')
BHLHE40	GGATCTCCTACCCGAACATCTCA	GAGCGAAAGTCCGCTGGATGACTG
ADAM19	CGAGAAGGTGAATGTGGCAGGA	AGCTCTGACACTGGATCTTCCC
CREB5	GTCAGTGAACTCCAGCATCATGG	GTGGTGAGTCAATGCAGCCTTC
KLF7	GAGTGGACAGAGCGACAGTGAC	CCTTTAGACACTAGCCGATGCCATG
NOTUM	GAGATCATCATCCGGAGCCAC	TCATCTCTTGCCCCGTGAAC
SNAI2	ATCTGCGGCAAGGCGTTTTCCA	GAGCCCTCAGATTTGACCTGTC
GAPDH	GAGCCACATCGCTCAGACACC	TGACAAGCTTCCCGTTCTCAGC

Supplementary Table 1. List of RT-PCR primers.

Region A	BHLHE40-2000f (GCTACTACTCTTCGCCCAGTCTC)	BHLHE40-1396r (CTGCCCTTGAACTTGCAGAACCTC)
Region A (nested)	BHLHE40-1946f (CCACAGCCAGGTCACTCAGCAGTG)	BHLHE40-1470r (GTATCGAGGCCACTGGCTGACAG)
Region B	BHLHE40-1419f (GAGGTTCTGCAAGTTCAAGGGCAG)	BHLHE40-1089r (CAGGGCGTGACGCTTCACGTGG)
Region B (nested)	BHLHE40-1391f (CGTGTCGGATTCATCAACTAGTGC)	BHLHE40-1141r (GAGCCTCTCTGCGTTGACCCAG)
Region C	BHLHE40-967f (GGTATCTTTCTGCGCTTGACTGG)	BHLHE40-447r (CAGGGAAATGAAGTAAGTTCCCGTC)
Region C (nested)	BHLHE40-831f (ACACCCTCCACGGTCAGGTGC)	BHLHE40-507r (CGTCTGACTCAAGCCGGGAGAG)
Region D	BHLHE40-469f (CGGGAACTTACTTCATTTCCCTGG)	BHLHE40-78r (GCAAGCCGAGGAGTAATGGAGAG)
Region D (nested)	BHLHE40-373f (CACCCAACTGGGCAGGACCCAG)	BHLHE40-122r (GAGCGAGTGGGTGGGTTGGAGC)

Supplementary Table 2. Primer pairs employed for chromatin immunoprecipitation assays.

SUPPLEMENTARY FIGURES

-2000 GCTACTACTCTTCGCCCAGTCTCGGCAGTGACCCCATTTTTTTTTCAATCCTTCCCACAGCCAGGTCACTCAGCAGTGGG

-1920 GTGACTGACCCGAAGCGCAGTGGGAAGGGCTTACAGTCATCTCTCAACTCCCCGAACTTGCAGGGCAGGGGAGCGCAGAG

-1840 GTCAGTGAAATCCCCAGCCAGGCCTTATTTTAGTCATGAGTTTCCACTGGCTCCCTCCTCTGACTGTATCAGCGCTGCTC

-1760 ACACCGCGGGATTGGAGTTTCAGCTGTTTTCCCTGCTCCCGGCTGAGTGAGAAGGTCACAGAGCCCGGACCTTTCCGGGG

-1680 GAGGTGGGTGGGGCCCTCCGGCAGCCTAAGTGAGATTGCAGAGATCTGGGGCCGCTGGAGGAGGTCCTTCCGCCCCGGGC

-1600 TTGGGGCTTTGGGTTAGCTTCTCCCCCGCTTCTCTACCTAGGCATCTCGGTTACAATGAAATTAAAAACAAAACACAAAG

-1520 CAACACAAGTTCTCATTGTTTTTCCCCTCTGTCAGCCAGTGGCCTCGATACAATTTTCCCAGGGCTGCCTTTCCTTTATT

-1440 GCTCAATTTAACCTCTTCCTAGAGGTTCTGCAAGTTCAAGGGCAGGATCCGTGTCGGATTCATCAACTAGTGCAGCGCCT

-1360 AGTGCAGAGCAGGGGGCTCAAGAAATACGTGTCGACCGCATGATTGCTAAGATTTCCCTGTAACAGGCGGTTTTTTTTTC

-1280 TTGTCTCTCTCATCCCTCCTGCCCTGGGTGCAGACCGCGAACAGCGCCGCCGAACTTCCCAACAGGCAGGGAGAGGGCCG

-1200 ATCCGGGCTGGCACGCCACGTCCCCGGGGTCTCAGTCCCTGGGTCAACGCAGAGAGGCTCGCGGTCCGCGGTGCTACGAC

-1120 CTGAGCCCGGCCACGTGAAGCGTCACGCCCTGCGCTGCTGCAGGGCAGGCGCCGCCGCCGCGGTTGTGTAACGCTGCAGC

-1040 CGGAGGGGAGGCGACCGGGGCGCCTCTGCTCCGGAGGGGGCAGAGGAGGGAGGGAGCGCCGGGCACAGCGCCCGGTATCT

-960 TTCTGCGCTTGACTGGCCGGGAGGAAGGGGGTGACACTGGGGCACCCAGGGGGCTGCGCAACCGGGTGCCCGCCCGCCCG

-880 CAGGTTGTGCCCAGGAGCGGAGCTCCTGGGAGCGAGGGGCCCTCGGCTCACACCCTCCACGGTCAGGTGCGCGCGGCGTG

-800 CTCGGCGGCAGCCTCAGCTGCCCCTGCGCTTGGCCGTCGGCCCGCTTCCCATGGGGTGACATCCGCCCCGCCCCTCGGTC

-720 CCTCCCCAAGGCGGGCAATTCCTGGACGCGAGGGTGAGCAGTGGGGGCAGGGAAGCCAGGACGGAAAGAAACCCCAGCCT

-640 CTGGGGAAGGCTGGGGGGCCGGGCGACTCCCTCCGCAGCGCGCCGGTTCCTCCAAGCGGGCCGGGCGGGGGAGGAGGAAG

-560 AGGGCTGGGCTGGAGCTAGCAAGGGGATATTCCTCTCCCGGCTTGAGTCAGACGCGGGCGGATCCGTCCTCCCCCGTTCC

-480 CTCCCAGGAGACGGGAACTTACTTCATTTCCCTGGGGCAGGTTCGCCCACGTTACCAACTTCTCCCCCTCCCCCAGCACC

-400 CCCGTCCCTTCCAGCTTCCGCGCCCCCCACCCAACTGGGCAGGACCCAGGTCGTGCTGCCACCCCCTCTTCGGGGAAAGG

-320 CGGCCGCAGCCGCAGACACCTGGGGGCCGGGGCTGGGGGTGGGGGCTCCCTAGCAGCCGCCGGAGCGTTGTCCAACACGT

-240 GAGACTCATGTGATGAAGCCGGGGGAGGGCGGGCAGGTCGCTCCTTCCCTCCCCGGCAGTGGCCAGACGTGCCTGGAGTC

-160 ACAGGGTAGAACACGTAGCTCCAACCCACCCACTCGCTCCCATTTAACCCAGCCCGCAGCCTCTCCATTACTCCTCGGCT

-80 TGCCCCCCCACCCCCCCACTCCGCCCTAACCGCCCCCCCCTCACCCGCTCCCTCCCGCCTCCCCGCCCGCCCCACTTCTC

+1 ATTCACTTGGCTCGCACGGCGCAGACAGACCGCGCAGGGAGCACACACCG

Supplementary Figure 1. 2000 bp of upstream promoter sequence and the first 50 transcribed nucleotides (in bold type) of the human *BHLHE40* gene (NCBI Reference Sequence NM_003670.3). 39 ETS core binding sequences (5'-**GGA**$^{\mathbf{A}}/_{\mathbf{T}}$-3', or $^{\mathbf{A}}/_{\mathbf{T}}$**TCC** in reverse), to which ETV1 may potentially bind, are highlighted in yellow color; please note that -1396 to -1391 encompasses two antiparallel, overlapping ETS sites. The consensus binding sequence for human ETV1 is 5'-$^{A}/_{G}$ $^{C}/_{G}$ $^{C}/_{A}$ **GGA** $^{\mathbf{A}}/_{\mathbf{T}}$ $^{G}/_{A}$ $^{T}/_{C}$ -3', as published by Wei *et al* [1].

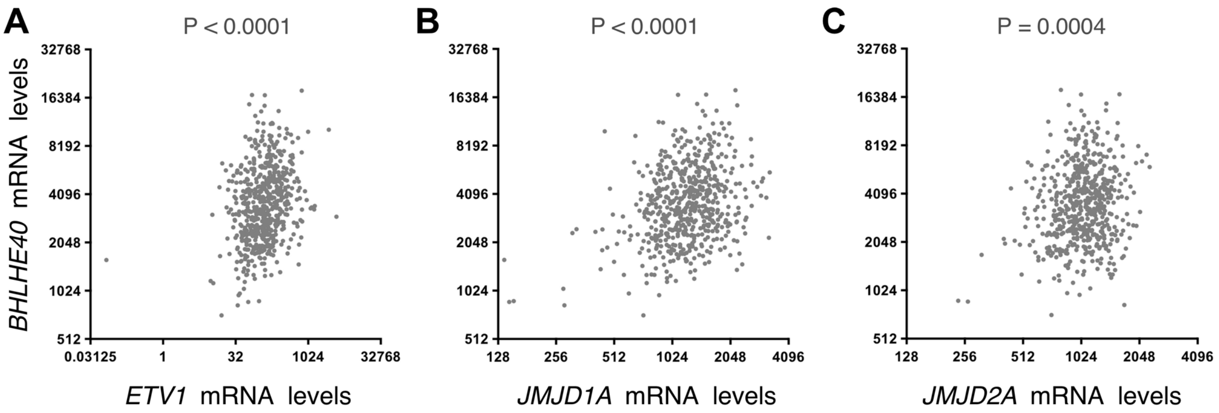

Supplementary Figure 2. Correlation of *BHLHE40* mRNA levels with those of (**A**) *ETV1* (r = 0.2772), (**B**) *JMJD1A* (r = 0.2569) or (**C**) *JMJD2A* (r = 0.1439) in 592 colorectal adenocarcinomas. Data were derived from the TCGA PanCancer Atlas (RSEM values; batch normalized from Illumina HiSeq_RNASeqV2) with determination of Spearman correlation coefficient (r).

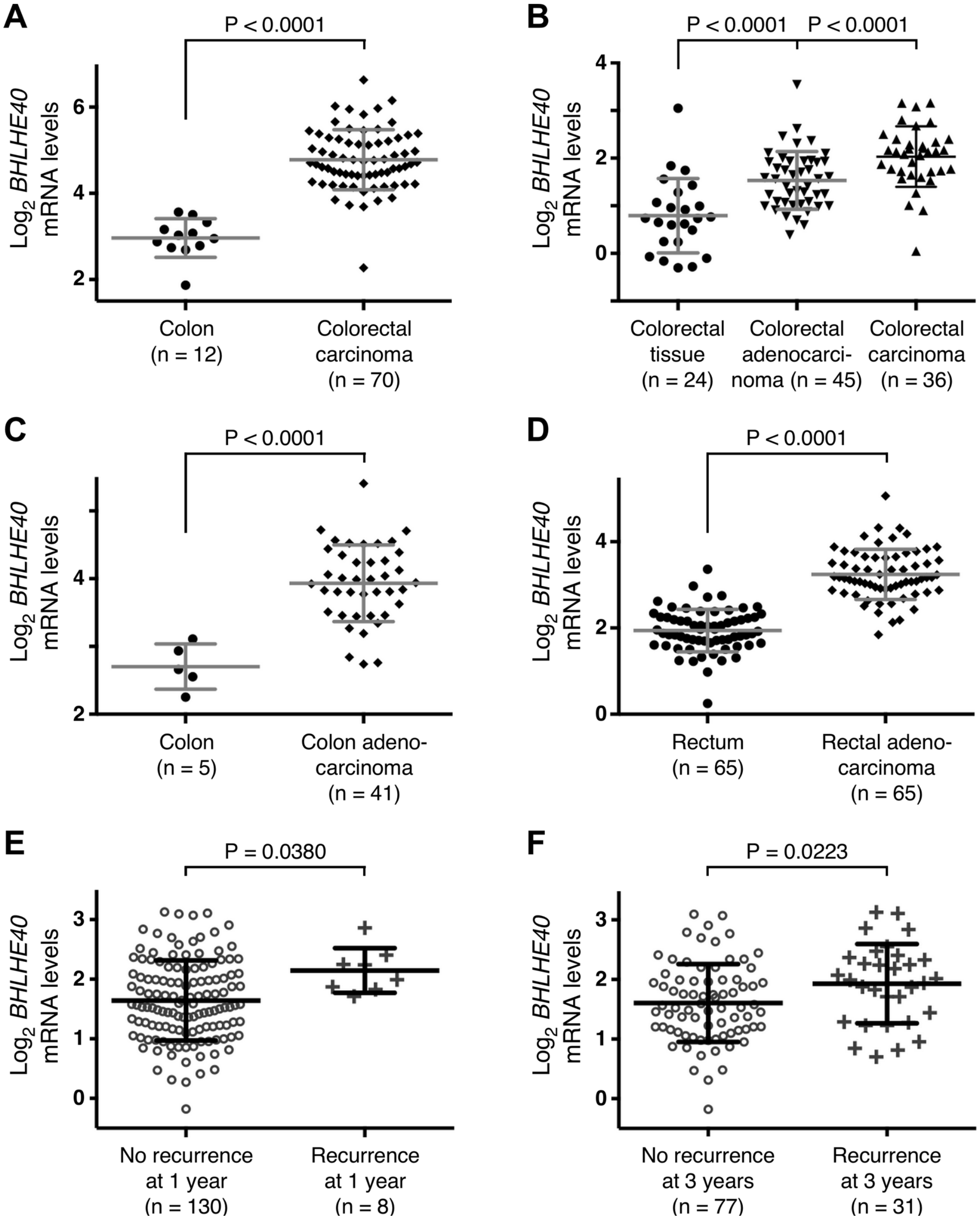

Supplementary Figure 3. Expression of *BHLHE40* mRNA (log2-median centered ratio) in published microarray data sets. (**A**) Data from Hong *et al* [2] with reporter 201170_s_at. (**B**) Data from Skrzypczak *et al* [3] with reporter 201169_s_at. (**C**) Data from Kaiser *et al* [4] with reporter 201170_s_at. (**D**) Data from Gaedcke *et al* [5] with reporter A_24_P268676. (**E**) and (**F**) Data from Smith *et al* [6] with reporter 201169_s_at. One-way ANOVA (Dunnett's multiple comparisons test) was used for panel B and an unpaired, two-tailed t test for all other panels.

SUPPLEMENTARY REFERENCES

1. Wei GH, Badis G, Berger MF, Kivioja T, Palin K, Enge M, Bonke M, Jolma A, Varjosalo M, Gehrke AR, Yan J, Talukder S, Turunen M, Taipale M, Stunnenberg HG, Ukkonen E, Hughes TR, Bulyk ML, Taipale J (2010) Genome-wide analysis of ETS-family DNA-binding in vitro and in vivo. *EMBO J.* **29**, 2147-2160.
2. Hong Y, Downey T, Eu KW, Koh PK, Cheah PY (2010) A 'metastasis-prone' signature for early-stage mismatch-repair proficient sporadic colorectal cancer patients and its implications for possible therapeutics. *Clin. Exp. Metastasis* **27**, 83-90.
3. Skrzypczak M, Goryca K, Rubel T, Paziewska A, Mikula M, Jarosz D, Pachlewski J, Oledzki J, Ostrowski J (2010) Modeling oncogenic signaling in colon tumors by multidirectional analyses of microarray data directed for maximization of analytical reliability. *PLoS One* **5**, e13091.
4. Kaiser S, Park YK, Franklin JL, Halberg RB, Yu M, Jessen WJ, Freudenberg J, Chen X, Haigis K, Jegga AG, Kong S, Sakthivel B, Xu H, Reichling T, Azhar M, Boivin GP, Roberts RB, Bissahoyo AC, Gonzales F, Bloom GC, Eschrich S, Carter SL, Aronow JE, Kleimeyer J, Kleimeyer M, Ramaswamy V, Settle SH, Boone B, Levy S, Graff JM, Doetschman T, Groden J, Dove WF, Threadgill DW, Yeatman TJ, Coffey RJ, Jr., Aronow BJ (2007) Transcriptional recapitulation and subversion of embryonic colon development by mouse colon tumor models and human colon cancer. *Genome Biol.* **8**, R131.
5. Gaedcke J, Grade M, Jung K, Camps J, Jo P, Emons G, Gehoff A, Sax U, Schirmer M, Becker H, Beissbarth T, Ried T, Ghadimi BM (2010) Mutated KRAS results in overexpression of DUSP4, a MAP-kinase phosphatase, and SMYD3, a histone methyltransferase, in rectal carcinomas. *Genes Chromosomes Cancer* **49**, 1024-1034.
6. Smith JJ, Deane NG, Wu F, Merchant NB, Zhang B, Jiang A, Lu P, Johnson JC, Schmidt C, Bailey CE, Eschrich S, Kis C, Levy S, Washington MK, Heslin MJ, Coffey RJ, Yeatman TJ, Shyr Y, Beauchamp RD (2010) Experimentally derived metastasis gene expression profile predicts recurrence and death in patients with colon cancer. *Gastroenterology* **138**, 958-968.
